# Individual differences in adolescent cortical development are associated with neighborhood characteristics: Longitudinal findings from the ABCD study

**DOI:** 10.1093/cercor/bhag034

**Published:** 2026-04-07

**Authors:** Chloe Carrick, Divyangana Rakesh, Lea Michel, Kathryn Bates, Delia Fuhrmann

**Affiliations:** Department of Psychology, Institute of Psychiatry, Psychology, and Neuroscience, King’s College London, Guy's Campus, Great Maze Pond, London SE1 1UL, United Kingdom; Neuroimaging Department, Institute of Psychiatry, Psychology, and Neuroscience, King’s College London, Centre for Neuroimaging Sciences, De Crespigny Park, Camberwell, London SE5 8AF, United Kingdom; Cognitive Neuroscience Department, Radboud University Medical Center, Nijmegen, The Netherlands; Department of Psychology, Institute of Psychiatry, Psychology, and Neuroscience, King’s College London, Guy's Campus, Great Maze Pond, London SE1 1UL, United Kingdom; Department of Psychology, Institute of Psychiatry, Psychology, and Neuroscience, King’s College London, Guy's Campus, Great Maze Pond, London SE1 1UL, United Kingdom

**Keywords:** ABCD study, adolescence, cortical development, individual differences, neighborhood characteristics

## Abstract

Developmental trajectories of adolescent cortical structure differ between individuals. Neighborhood environments are increasingly recognized as influencing this variability. Few studies have examined how multifaceted neighborhood contexts relate to individual changes in cortical maturation patterns. Using 3 waves of neuroimaging data from the ABCD study (*n* = 11,639 with at least one scan), and latent growth models, the present investigation examined associations between exposure to neighborhood disadvantage and educational, health, and environmental opportunities at ages 9 to 10, and interindividual variability in trajectories of cortical thickness and surface area development between ages 9 and 15. Individuals exposed to disadvantaged neighborhoods showed lower cortical thickness and surface area, and accelerated rates of change in these metrics across adolescence, whereas greater neighborhood opportunities were associated with higher cortical thickness and surface area and a slower pace of change. Our findings indicate interindividual variability in cortical maturational trajectories and provide evidence for the role of neighborhood environments, including positive and negative features, in shaping this variability. This emphasizes the need for future studies examining multiple facets of neighborhood ecologies when examining their influence on adolescent cortical development.

## Introduction

Adolescence is characterized by extensive changes to cortical gray matter, including nonlinear decreases in surface area and thickness (Mills et al. 2016; Vijayakumar et al. 2016; Tamnes et al. 2017). A growing body of literature indicates that there is considerable interindividual heterogeneity in cortical structure development (Becht and Mills 2020; Mills et al. 2021; Fuhrmann et al. 2022a; Bottenhorn et al. 2023, 2024; Carrick et al. 2025). These individual differences are thought to be driven, in part, by environmental factors, including proximal family and school settings, as well as broader social and structural factors at the neighborhood level and beyond (Ferschmann et al. 2022; Choudhury et al. 2023; Polemiti et al. 2024). There is a need for studies that not only quantify individual differences in cortical development but situate them within their ecological context (Ferschmann et al. 2022). This work can enhance our understanding of how external factors shape variability in patterns of structural brain development across adolescence (Choudhury et al. 2023; Whittle et al. 2024).

It has been hypothesized that exposure to stressful environmental experiences early in life can become neurobiologically embedded and result in lasting changes to brain architecture (Tooley et al. 2021; Whittle et al. 2024; Nelson et al. 2025). A key environmental influence associated with variability in cortical development is socioeconomic status (SES), a construct comprising access to financial and nonfinancial resources (Farah 2017; Long and Renbarger 2023). Research suggests that variability in SES is associated with individual differences in cortical maturation patterns (Rakesh et al. 2023a). While studies investigating this association have primarily measured SES at the family level, using indicators such as parental income and education, less is known about the influence of neighborhood socioeconomic characteristics on cortical development (Rakesh et al. 2021a).

Neighborhoods have been increasingly recognized as an important developmental context (Lewis et al. 2025). Growing up in a neighborhood characterized by socioeconomic disadvantage is associated with a myriad of developmental outcomes including worse physical health, mental health, and academic achievement (Leventhal and Dupéré 2019; Shoari et al. 2025; Thomas et al. 2025). Cross-sectional research has begun to link neighborhood disadvantage to individual differences in cortical thickness and surface area in adolescence (Parker et al. 2017; Vargas et al. 2020; Hackman et al. 2021; Tomasi and Volkow 2021; Miller et al. 2022; Rakesh et al. 2022; Nolte et al. 2024), even after accounting for family-level SES influences (Miller et al. 2022; Rakesh et al. 2022). This suggests that neighborhood environments play an important role in shaping individual variation in adolescent cortical structure, but few longitudinal studies have examined the relationship between neighborhood disadvantage and cortical development over time.

A recent study using data from the Adolescent Brain and Cognitive Development (ABCD) cohort indicated that neighborhood deprivation was associated with cortical volumetric decline across ages 9 to 13 (Thomas et al. 2025). Furthermore, some work using longitudinal data from Australia indicates that neighborhood disadvantage was associated with brain maturation from early to late adolescence (Whittle et al. 2017; Rakesh et al. 2021a). However, these latter studies were conducted in the same sample of 166 adolescents, indicating the need for additional investigations of this association in large, socio-demographically diverse samples, covering a wide age span. Thus, given limitations in extant literature, it remains unclear how neighborhood disadvantage is related to patterns of cortical maturation across adolescent development.

Several theoretical models have been proposed to explain how stressful experiences, such as exposure to socioeconomic disadvantage, could influence the pace of cortical development (for an overview, see Rakesh et al. 2023a). Evolutionary-based frameworks such as the stress acceleration hypothesis (Callaghan and Tottenham 2016) often link stressful experiences to accelerated development. This theory posits that exposure to stressful environments early in life may accelerate the pace of neurodevelopment as an adaptive response, facilitating earlier maturation, whereas early exposure to enriching environments may contribute to protracted neurodevelopment, promoting a prolonged period of neuroplasticity (Tooley et al. 2021). Consistent with this perspective, potentially stressful environmental exposures such as the COVID-19 pandemic (Van Drunen et al. 2023), as well as household and neighborhood deprivation (Thomas et al. 2025; Tsomokos et al. 2025), have been associated with faster cortical development, while cognitively enriching experiences, such as musical practice, have been associated with a protracted pace of change (van Drunen et al. 2026). However, evidence is mixed for the direction of the association between stressful experiences and the pace of cortical maturation. These discrepancies might be explained by differences in the characteristics of the stressful experience (e.g., whether it is classified as threat or deprivation) and different contextual factors (e.g., presence of social support) that may shape the pace of development differently (Colich et al. 2020; Gee 2021; Ellis et al. 2022). For instance, some evidence suggests that exposure to threatening environments is associated with accelerated neurodevelopment, whereas deprivation is associated with delayed maturation (Beck et al. 2025). However, other studies report the opposite pattern; for instance, Keding et al. (2021) found that adolescent girls exposed to physical neglect showed accelerated brain development, while those who experienced abuse exhibited delayed maturation. Additionally, accelerated brain development is not uniquely associated with stressful experiences. For instance, Becht et al. (2021) found that increased friendship quality was associated with accelerated cortical thinning*.* A recent review indicates that evidence most consistently supports an association between socioeconomic disadvantage and an atypical cortical maturation trajectory marked by reduced cortical surface area, thickness, and volume and slower change over time (Rakesh et al. 2023a). However, the authors note that few studies have specifically examined the relationship between neighborhood socioeconomic disadvantage and cortical development. Thus, given a lack of empirical work examining this association, it is unclear how existing theoretical frameworks apply to it.

It is further worth noting that studies investigating the relationship between neighborhoods and brain development, both cross-sectional and longitudinal, have predominantly focused on socioeconomic disadvantage only. These studies often rely on composite measures reflecting education, income, and employment levels, such as the Area Deprivation Index (Singh 2003). It is increasingly recognized that solely focusing on disadvantage risks reinforcing a deficit-based approach, overlooking positive aspects of the environment that can promote resilience to stressful, adverse experiences (DeJoseph et al. 2024; Zhou et al. 2025). A recent systematic review indicates that different neighborhood characteristics, encompassing both positive and negative factors, have distinct effects on cortical structure, underscoring the need for studies that include comprehensive assessments of neighborhood contexts (Lewis et al. 2025). Additionally, recent cross-sectional work harnessing ABCD data found that 9- to 10-year-olds living in high-opportunity neighborhoods had larger cortical volume, thickness, and surface area, which remained after controlling for neighborhood disadvantage, and household indicators of SES (Zhou et al. 2025). Broadening the lens through which we investigate neighborhood contexts is crucial for understanding the potential positive, as well as negative impacts of the neighborhood on adolescent brain development. However, longitudinal research examining how positive neighborhood features relate to individual trajectories of structural brain maturation across adolescence remains limited.

We leveraged longitudinal data from the ABCD study (Casey et al. 2018) to characterize interindividual variability in adolescent cortical development and to test how neighborhood environments (disadvantage, educational opportunity, and health and environmental opportunity) relate to this variability. Our hypotheses were preregistered prior to data access (https://osf.io/qg3fh). We hypothesized that individuals would differ in the rate of change of cortical development. We further hypothesized that these individual differences would manifest as distinct subgroups of cortical development, defined by variation in baseline cortical structure (intercept) and rate of change (slope). Additionally, we hypothesized that neighborhood characteristics would be associated with distinct patterns of cortical development. Specifically, we expected that subgroups of cortical maturation would differ in levels of (i) neighborhood disadvantage, (ii) educational opportunity, and (iii) health and environmental opportunity.

Importantly, there is a dearth of longitudinal research examining (i) the extent of interindividual variability in cortical maturation and (ii) how multifaceted neighborhood environments shape the pace of this development. Given this lack of evidence, we did not have specific hypotheses about the number of subgroups that would be identified, nor the patterns of development in each subgroup. Furthermore, in light of mixed evidence regarding the influence of potentially stressful versus enriching experiences, such as neighborhood disadvantage or opportunities, on the pace of neurodevelopment (Becht et al. 2021; Keding et al. 2021; Tooley et al. 2021; Van Drunen et al. 2023, 2026; Rakesh et al. 2023a; Beck et al. 2025), we did not have specific directional hypotheses regarding the association between neighborhood characteristics and cortical maturation. Considering the importance of neighborhood contexts for healthy development, we hope to address this research gap and elucidate how multifactorial neighborhood settings are associated with global metrics of cortical maturation across adolescence.

## Materials and methods

### Cohort and study design

Participants are drawn from the prospective, longitudinal Adolescent Brain and Cognitive Development study (ABCD). At baseline, ABCD included data for ~ 11,800 9- to 10-year-olds, recruited from 21 sites across the United States (Garavan et al. 2018). The present investigation includes data from the baseline, 2-year, and 4-year follow-up assessments (referred to here as imaging timepoints 1, 2, and 3) of Data Release 5.1 (https://nda.nih.gov/abcd). Our final sample included those who had imaging data for at least one of the three imaging timepoints. After quality control procedures (see the Measures section), this included *n* = 11,639 participants (2,446 with three scans, 5,635 with two scans, and 3,558 with one scan; See Table 1 for participant demographic information and Supplementary Table 1A and 1B for participant demographic characteristics at each timepoint, for those with only 1 timepoint, and those with 2 or more timepoints). Participants were, on average, 9.92 years at neuroimaging timepoint 1, 11.96 years at timepoint 2, and 14.08 years at timepoint 3. Descriptions of missingness are reported in Supplementary Table 2.

**Table 1:** Participant demographic information.

**Demographic characteristic**	**Value**
**Sex**	***N* (%)**
Male	6,017 (51.70%)
Female	5,547 (47.66%)
Intersex-Male	<10
**Ethnicity**	***N* (%)**
White	5,900 (50.69%)
Hispanic	2,301 (19.77%)
Black	1,656 (14.22%)
Asian	237 (2.04%)
Other	1,174 (10.09%)
**Neighborhood characteristics**	**M (SD) [range]**
Neighborhood disadvantage	−0.01 (0.84) [−1.49, 3.92]
Educational opportunity	60.41 (29.69) [1,100]
Health/environmental opportunity	58.73 (30.12) [1,100]
**Income-to-needs ratio**	3.72 (2.46) [0.03, 12.32]

*Note.* M, mean; SD, standard deviation.

### Measures

#### Cortical development

3T MRI scans were acquired by ABCD using seven different imaging device models (Prisma, Prisma fit, Discovery MR750, Achieva dStream, Ingenia, SIGNA Premier, and SIGNA UHP) manufactured by three imaging device manufacturers (Siemens, GE Medical Systems, and Philips Medical Systems). Two indices of whole-brain cortical development were examined in this study: (i) mean cortical thickness and (ii) total cortical surface area. These estimates, defined by the Desikan–Killiany atlas, were derived from cortical surface reconstruction from T1w images (see Hagler et al. 2019 for further information on imaging processing and analysis). Imaging data were excluded based on quality control procedures recommended by ABCD (imgincl_t1w_include = 0; https://nda.nih.gov/data-structure/abcd_imgincl01). Data from 633 individuals (688 observations) were excluded, resulting in a final sample of 22,166 observations from 11,639 participants.

#### Neighborhood characteristics

Descriptive statistics for participant scores in neighborhood characteristics are included in Table 1 (see Supplementary Table 2 for descriptions of missingness). Density plots and correlations between neighborhood disadvantage, neighborhood educational opportunity, and neighborhood health/environmental opportunity are presented in Supplementary Fig. 1.

##### Neighborhood disadvantage

In line with previous studies conducted in the ABCD cohort (Taylor et al. 2020; Ku et al. 2024), neighborhood disadvantage was measured at baseline (mean age 9.91 years) using nine items from the Area Deprivation Index (ADI), a 17-item measure reflecting neighborhood disadvantage characteristics corresponding to the participant’s primary address (Singh 2003; Kind et al. 2014; see the list of items in Supplementary Table 3). Consistent with previous research (Taylor et al. 2020), scores on these nine items were converted to *z*-scores, and three items were then reverse-scored (percentage of homeowners, percentage of population aged ≥25 years with at least a high school diploma, median family income) so that higher scores on these items reflected higher levels of disadvantage. Items were then averaged, with higher scores on this metric indicating higher neighborhood disadvantage. Other items assess poverty level (i.e., percentage of families below the poverty level, and the percentage below 138% of the poverty threshold), income disparity, employment (labour force percentage aged >16 and unemployed), the percentage of single-parent households, and homes without a motor vehicle in the participant’s neighborhood.

##### Educational, health, and environmental opportunities

The Childhood Opportunity Index (COI) 2.0 was used to assess neighborhood educational and health and environmental opportunities (Ferrara et al. 2024). This metric assesses neighborhood characteristics across three domains (education, health and environment, and social–economic variables). The (i) education and (ii) health and environment (referred to hereinafter as health/environment) domain scores were used in the present investigation. Items in these domains capture access to educational resources (e.g., high-quality early childhood educational centres), health insurance, green space, and other environmental factors (e.g., exposure to pollutants and heat). Prior research suggests that the COI is a valid measure of multidimensional neighborhood contexts, demonstrating associations with childhood developmental outcomes (Ferrara et al. 2024; Zhou et al. 2025). Higher COI scores represent higher levels of neighborhood opportunity.

#### Household income-to-needs ratio

Following the approach of prior investigations using ABCD data (Rakesh et al. 2022, 2023b, 2024), the household income-to-needs ratio was calculated by dividing the median value of the participant’s household income band by the federal poverty line for their family size (descriptive information in Table 1 and Supplementary Table 1A and 1B). The income-to-needs ratio was included as a confounder in adjusted analyses (see the Adjusted Analyses section).

### Statistical analyses

All analyses were conducted in R version 4.3.1 (R Core Team, 2024). The preregistration and analysis scripts for the analyses described below are provided on the Open Science Framework (https://osf.io/avu29/).

#### Examining trajectories of cortical development: latent growth models

In line with our preregistration plan, latent growth models were used to identify average trajectories of cortical thickness and surface area development and to test for interindividual variability in these trajectories. Latent growth models capture developmental change by treating model parameters (i.e., the intercept and slope) as latent variables, using factor loadings to indicate their relationship to the observed timepoints (Duncan and Duncan 2009; Grimm et al. 2011). We fit separate latent growth models to (i) cortical thickness and (ii) cortical surface area, capturing change using a latent basis slope (i.e., fixing the factor loading for the first timepoint to 0, the third timepoint to 1, and freely estimating the factor loading for the second timepoint). This approach can thus model development without specifying its expected shape (McNeish 2019). For both cortical thickness and surface area, we examined (i) the slope to assess the average direction of change and (ii) the variance of the intercept and slope, to determine whether there were individual differences in trajectories of cortical development.

Before fitting latent growth models to model cortical thickness and surface area development, we transformed the data by multiplying cortical thickness values by 10 and dividing surface area values by 10,000. This approach was necessary to facilitate model convergence. To additionally support model convergence, the residual variances were constrained to be equal across timepoints. Model fit was compared between a latent basis growth model and a linear growth model using a Likelihood Ratio Test and comparing the Akaike Information Criterion between both models (AIC; Akaike 1998), with lower AIC values indicating better model fit.

Latent growth models were fit using the *lavaan* package (version 0.6-19; Rosseel 2012). Models were fit using a maximum likelihood estimator, with robust standard errors, and Satorra–Bentler scaled test statistics. Full information maximum likelihood (FIML) was used to estimate missing data in models; this method is considered a valid approach for estimating missing data in structural equation models, demonstrating less bias than other techniques for estimating missing data, such as imputation (Enders and Bandalos 2001). Model fit was assessed using the *χ*^2^ test, the Root Mean Square Error of Approximation (RMSEA), the Comparative Fit Index (CFI), and the Standardized Root Mean Square Residual (SRMR). Models were defined as having good fit if CFI > 0.97, SRMR < 0.05, and RMSEA < 0.05. Acceptable fit was defined as CFI = 0.95 to 0.97, SRMR = 0.05 to 0.10, and RMSEA 0.05 to 0.08 (Schermelleh-Engel et al. 2003). We characterized standardized parameter estimates above 0.10 as small effects, between 0.20 and 0.30 as typical, and over 0.30 as large effect sizes (Gignac and Szodorai 2016).

#### Assessing subgroups of cortical development: growth mixture models and k-means clustering

As preregistered, growth mixture models were next used to test for the presence of subgroups of individuals with distinct patterns of cortical development, fit using the *lcmm* package in R (version 2.21; Proust-Lima et al. 2017). Separately for cortical thickness and surface area, we fit four growth mixture models with an increasing number of subgroups (1 to 4) to determine the optimal number of subgroups of cortical development (Wardenaar, 2020). Given issues with growth mixture model estimation (described in detail in the Subgroups of Cortical Development Showed Parallel Trajectories section), and in line with our preregistered plan, we proceeded to use *k*-means clustering, an unsupervised machine learning technique, to identify subgroups of cortical development. Models were fit using the *kml* package (version 2.5.0; Genolini et al. 2015). As per the default recommendation, we examined 2 to 6 cluster solutions (Genolini and Falissard 2011). Findings using both growth mixture models and *k*-means clustering indicated that there were no distinct subgroups of cortical surface area or thickness development (see the Subgroups of Cortical Development Showed Parallel Trajectories section).

#### Association between neighborhood characteristics and individual differences in cortical development

**Figure 1:**
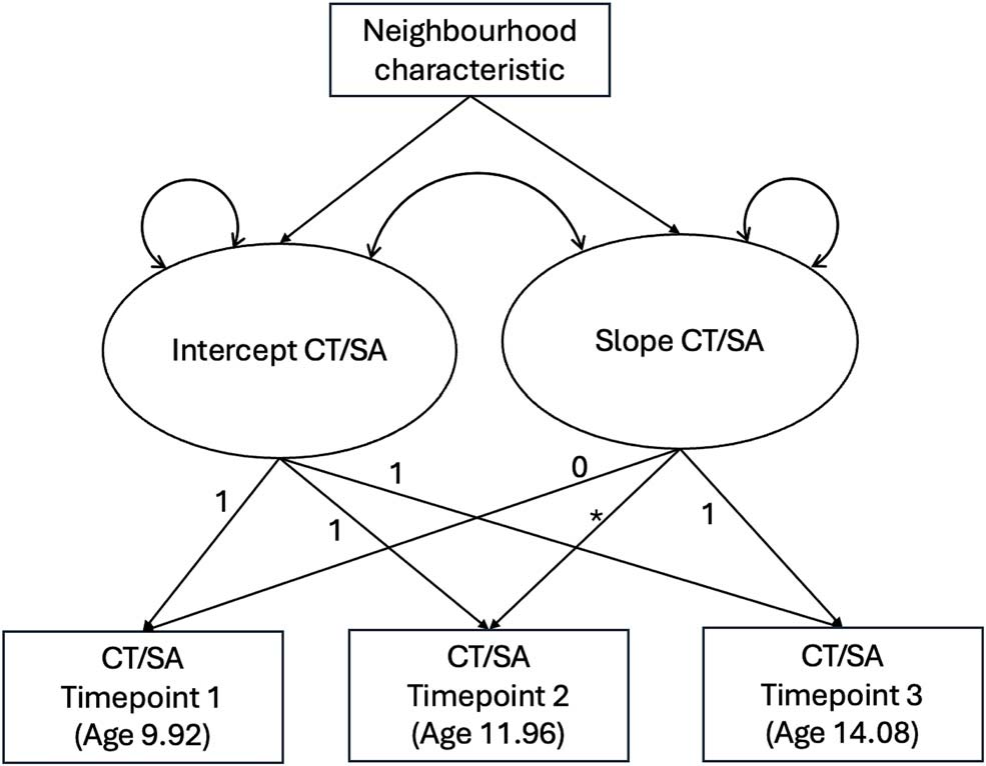
Schematic path diagram depicting latent growth models of cortical thickness and surface area development, with neighborhood characteristics as predictors of the intercept and slope of cortical development. Average age is presented at each timepoint. Double-headed arrows represent the slope and intercept variances and covariances. Numbers represent the factor loadings. *Factor loading was freely estimated. CT, cortical thickness; SA, cortical surface area.

As we did not identify distinct subgroups of cortical development in our analyses (see the Subgroups of Cortical Development Showed  Parallel Trajectories section), we were unable to examine the relationship between neighborhood characteristics and cortical maturation subgroup membership as originally preregistered. As a nonregistered follow-up, we instead tested for continuous associations. We employed latent growth models to investigate how neighborhood features were associated with individual differences in the intercept and slope of cortical development (see path diagram in Fig. 1). Separately for cortical thickness and surface area, we fit three latent growth models, one for each neighborhood metric. In each model, we estimated the regression paths from that neighborhood characteristic to the intercept and slope parameters. To correct for multiple comparisons between the three neighborhood characteristics and the intercept and slope of cortical development, we adjusted the alpha level of these regression paths with a Bonferroni correction (significance level = 0.05/3 = 0.017). When examining the influence of neighborhood factors on the intercept and slope of cortical surface area development, we transformed surface area values to the proportion of the maximum possible score (POMS) across timepoints (i.e., all values were scaled between 0 and 1). This approach was necessary to support model convergence and has been used in prior investigations employing latent growth models (Fuhrmann et al. 2022b; Little 2024).

#### Adjusted analyses

We adjusted our analyses to control for the potentially confounding effects of sex, scanner type, and income-to-needs ratio (see path diagram in Supplementary Fig. 2). These variables have been associated with individual differences in cortical developmental trajectories (Peper et al. 2020; Rakesh and Whittle 2021; Dudley et al. 2023). Scanner type (i.e., the imaging device model: Prisma, Prisma fit, Discovery MR750, Achieva dStream, Ingenia, SIGNA Premier) was included as a fixed categorical variable in analyses (Dudley et al. 2023). Scanner type and sex were dummy-coded in all models.

#### Specificity analyses

To assess the unique contribution of each neighborhood characteristic to cortical development, we conducted separate specificity analyses for cortical thickness and surface area, fitting a latent growth model with a regression path examining all three neighborhood factors as predictors of the slope and intercept of cortical development. This model was also adjusted for the influence of sex, scanner type, and income-to-needs ratio.

## Results

### Trajectories of cortical development decreased and were heterogeneous between individuals

**Figure 2:**
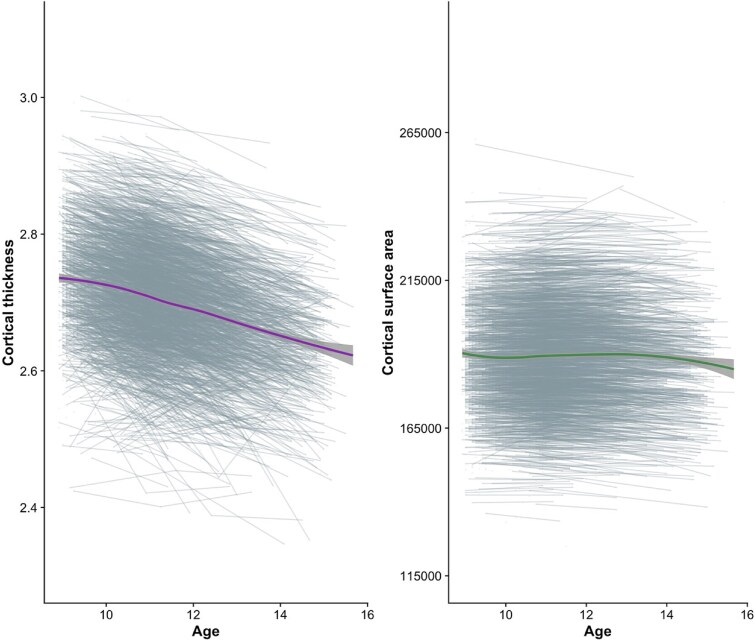
Changes in average cortical thickness (mm) and total cortical surface area (mm^2^) with age for a random subsample of 3,000 participants. Average developmental trajectories fit using loess with 95% confidence intervals are presented, overlaid on spaghetti plots of individual trajectories.

To understand average and individual-level changes in cortical maturation, we first fit separate latent growth models to (i) cortical thickness and (ii) cortical surface area development. These models demonstrated a good fit (Table 2). The latent growth model with a basis slope fit better than a linear growth model for cortical thickness (ΔAIC = 44; χ^2^(3) = 41.95, *P* < 0.001) and surface area (ΔAIC = 442.71; χ^2^(3) = 310.11, *P* < 0.001), indicating that change in these metrics was not linear across time. Significant, negative slope estimates indicated that, on average, in the sample, cortical thickness and surface area significantly decreased over time (Table 2; average trajectories visualized in Fig. 2). However, it is worth noting that, visually, the trajectory of surface area development does not show a clear decline; instead, it appears relatively stable until age 14, after which a decline becomes more apparent (Fig. 2).

**Table 2:** Latent growth model estimates for cortical thickness and surface area.

**Cortical thickness**					
**Model fit: χ^2^(2) = 18.54, *P* < 0.001; RMSEA = 0.03 [0.02 to 0.04]; CFI = 0.999; SRMR = 0.01**					
**Path**	**Estimate**	**SE**	***z*-value**	***P-*value**	**Std Est**
Intercept	27.27	0.01	3694.18	<0.001	37.14
Slope	−0.80	0.01	−100.70	<0.001	−3.51
Intercept variance	0.54	0.01	60.82	<0.001	1.00
Slope variance	0.05	0.01	4.88	<0.001	1.00
Intercept ~ ~ Slope	−0.02	0.01	−2.32	0.02	−0.10
**Cortical surface area**					
**Model fit: χ^2^(2) = 133.06, *P* < 0.001; RMSEA = 0.08 [0.07 to 0.09]; CFI = 0.997; SRMR = 0.01**					
**Path**	**Estimate**	**SE**	***z*-value**	***P*-value**	**Std Est**
Intercept	18.94	0.02	1132.22	<0.001	10.55
Slope	−0.17	0.01	−28.23	<0.001	−0.79
Intercept variance	3.22	0.04	74.80	<0.001	1.00
Slope variance	0.05	0.01	9.15	<0.001	1.00
Intercept ~ ~ Slope	0.11	0.01	9.78	<0.001	0.29

*Note*. Intercept ~ ~ Slope represents the covariance between the intercept and slope parameters. RMSEA, Root Mean Square Error of Approximation; CFI, Comparative Fit Index; SRMR, Standardized Root Mean Square Residual; SE, Standard Error; Std Est, Standardized Estimate.

There were individual differences in both baseline levels and patterns of change in cortical thickness and surface area, as indicated by significant variances in intercept and slope estimates (Table 2). Upon extracting individual slope estimates, we found that 337 individuals (2.89% of the sample) exhibited positive slopes for surface area development, indicating an increase in surface area across the investigated age range for these individuals. In the sections that follow, our interpretation of associations between the slope of surface area development and neighborhood factors pertains to the large majority of the sample, who showed decreases in surface area over this period.

### Subgroups of cortical development showed parallel trajectories

Growth mixture models were used to test for the presence of subgroups of cortical development. We first fit latent class growth analyses, a simpler type of growth mixture model in which all individuals within a subgroup are assumed to be homogeneous (i.e., no variance within each subgroup). Upon visualization of latent class growth analyses, we observed that the resulting trajectories in each subgroup were parallel, i.e., the slope of trajectories was not qualitatively distinct from one another (see Supplementary Fig. 3. Further model information is presented in Supplementary Table 4). When fitting growth mixture models with an increasing number of subgroups, we observed that there was no solution in which the proportion of the sample in each group was >5%; one of the criteria for determining whether there are meaningful subgroups of developmental trajectories (Jung and Wickrama 2007; Nguena Nguefack et al. 2020; see Supplementary Table 4). Our findings using *k*-means clustering similarly indicated that identified subgroup trajectories were parallel, ie not qualitatively distinct from one another (Supplementary Fig. 4). This prevented us from examining the relationship between neighborhood characteristics and subgroup membership, as planned. Given the significant slope variances in developmental trajectories of cortical thickness and surface area, we instead examined how neighborhood characteristics were continuously associated with individual differences in the sample.

### Neighborhood characteristics were associated with interindividual variability in cortical development

We fit three latent growth models, separately for cortical thickness and surface area; in each one, we examined the continuous association between a neighborhood characteristic (neighborhood disadvantage, educational opportunity, health/environmental opportunity) and variance in baseline levels (intercept) and change (slope) in cortical development. These latent growth models showed good model fit (Table 3).

**Table 3:** Latent growth model regression paths of neighborhood characteristics to cortical thickness and surface area development.

**Neighborhood disadvantage**						
**CT**	**Model fit:** χ^2^(3) = 19.43, *P* < 0.001; RMSEA = 0.02 [0.01 to 0.03]; CFI = 0.999; SRMR = 0.006					
	**Regression path**	**Estimate**	**SE**	***z*-value**	***P*-value**	**Std Est**
	Slope ~ ND	−0.03	0.01	−3.07	0.002	−0.12
	Intercept ~ ND	−0.13	0.01	−14.39	<0.001	−0.15
**SA**	**Model fit:** χ^2^(3) = 149.39, *P* < 0.001; RMSEA = 0.07 [0.06 to 0.07]; CFI = 0.996; SRMR = 0.005					
	**Regression path**	**Estimate**	**SE**	***z*-value**	***P*-value**	**Std Est**
	Slope ~ ND	−0.002	0.001	−3.77	<0.001	−0.13
	Intercept ~ ND	−0.03	0.001	−22.52	<0.001	−0.21
**Neighborhood educational opportunity**						
**CT**	**Model fit:** χ^2^(3) = 18.95, *P* < 0.001; RMSEA = 0.02 [0.01 to 0.03]; CFI = 0.999; SRMR = 0.006					
	**Regression path**	**Estimate**	**SE**	***z*-value**	***P*-value**	**Std Est**
	Slope ~ EO	0.001	0.0003	3.26	0.001	0.12
	Intercept ~ EO	0.003	0.0003	13.16	<0.001	0.14
**SA**	**Model fit:** χ^2^(3) =158.42, *P* < 0.001; RMSEA = 0.07 [0.06 to 0.08]; CFI = 0.996; SRMR = 0.005					
	**Regression path**	**Estimate**	**SE**	***z*-value**	***P*-value**	**Std Est**
	Slope ~ EO	0.0001	0.00002	4.02	<0.001	0.14
	Intercept ~ EO	0.001	0.00004	18.38	<0.001	0.18
**Neighborhood health/environmental opportunity**						
**CT**	**Model fit:** χ^2^(3) = 19.31, *P* < 0.001; RMSEA = 0.02[0.01 to 0.03]; CFI = 0.999; SRMR = 0.006					
	**Regression path**	**Estimate**	**SE**	***z*-value**	***P*-value**	**Std Est**
	Slope ~ HO	0.001	0.0003	4.21	<0.001	0.15
	Intercept ~ HO	0.003	0.0003	13.55	<0.001	0.14
**SA**	**Model fit:** χ^2^(3) = 159.12, *P* < 0.001; RMSEA = 0.07[0.06 to 0.08]; CFI = 0.996; SRMR = 0.005					
	**Regression path**	**Estimate**	**SE**	***z*-value**	***P*-value**	**Std Est**
	Slope ~ HO	0.0001	0.00002	4.10	<0.001	0.14
	Intercept ~ HO	0.001	0.00004	17.84	<0.001	0.18

*Note*. CT, cortical thickness; SA, cortical surface area; RMSEA, Root Mean Square Error of Approximation; CFI, Comparative Fit Index; SRMR, Standardized Root Mean Square Residual; SE, Standard Error; ND, neighborhood disadvantage; EO, neighborhood educational opportunity; HO, neighborhood health/environmental opportunity.

**Figure 3:**
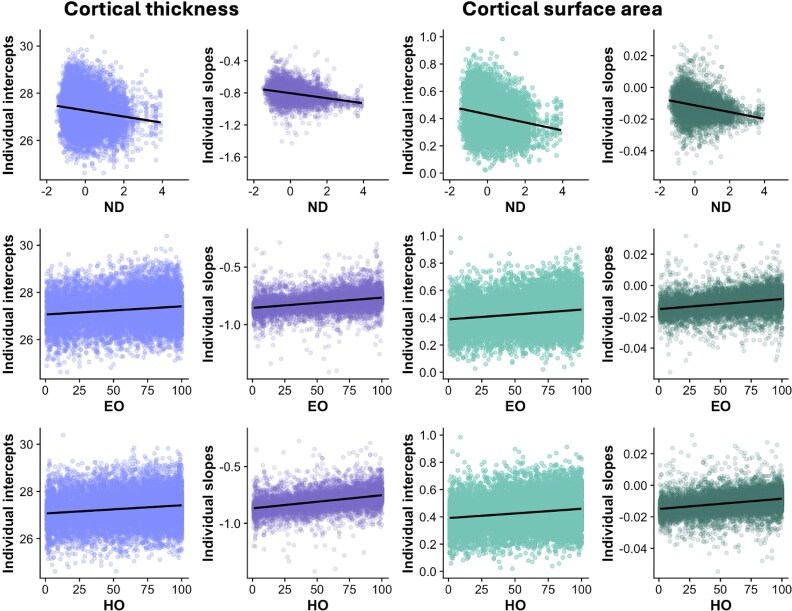
Relationship between neighborhood characteristics and individual intercept and slope values for cortical thickness (purple) and surface area (green). To support model convergence, thickness values were rescaled by multiplying by 10, and surface area values were scaled between 0 and 1. ND, neighborhood disadvantage; EO, neighborhood educational opportunity; HO, neighborhood health/environmental opportunity.

Overall, results indicated that each investigated neighborhood characteristic was significantly associated with the intercept and slope of cortical development (Table 3). Higher neighborhood disadvantage was associated with lower baseline levels of cortical thickness and surface area (intercepts) and faster decreases in these metrics across development (slopes). Higher educational and health/environmental opportunities in the neighborhood were associated with higher baseline levels of cortical thickness and surface area (intercepts) and slower declines in these metrics across development (slopes). Effect sizes for these relationships were small. The association between neighborhood characteristics and individual differences in the intercept and slope of cortical thickness and surface area development is presented in Fig. 3.

### Adjusted analyses: influence of sex, scanner type, and income-to-needs ratio

To control for the influence of sex, scanner type, and income-to-needs ratio on developmental trajectories of cortical thickness and surface area, these variables were included as covariates. Associations between neighborhood characteristics and cortical thickness and surface area development remained significant and in the same direction (Supplementary Table 5). Overall, these results indicate that neighborhood disadvantage, educational, and health/environmental opportunities account for unique variance in developmental trajectories of cortical thickness and surface area, beyond that explained by the investigated covariates. Effect sizes remained small after covariates were included in the model (Supplementary Table 5).

An additional sensitivity analysis was conducted, controlling for total intracranial volume to examine the extent to which differences in overall brain size accounted for the relationship between neighborhood characteristics and cortical development. Results for cortical surface area remained unchanged. For cortical thickness, the intercept remained significantly associated with all neighborhood characteristics, and the slope of development remained significantly associated with health and environmental opportunity. However, the slope of thickness development was no longer significantly associated with neighborhood disadvantage (*P* = 0.07) or educational opportunity (*P* = 0.05) after accounting for intracranial volume (Supplementary Table 6). These findings suggest that, for cortical thickness, the relationship between the pace of developmental change and exposure to neighborhood educational and disadvantage characteristics may be primarily driven by differences in global brain size.

### Specificity analysis: unique influence of each neighborhood characteristic

To assess the unique explanatory value of each neighborhood characteristic to cortical development, we conducted separate specificity analyses for cortical thickness and surface area (Supplementary Table 7). First, we fit a latent growth model for each cortical metric that included a regression path with all three neighborhood characteristics as predictors of the intercept and slope of that cortical metric. Second, we repeated this, controlling for sex, scanner type, and income-to-needs ratio in each model.

These models suggested that, when considering neighborhood disadvantage and opportunity indices together, only neighborhood opportunities explain unique variance in the pace of cortical thickness development, while both neighborhood disadvantage and opportunities are related to baseline levels of cortical thickness and surface area (Supplementary Table 7). Although neighborhood characteristics were correlated, these values were below commonly accepted thresholds for problematic multicollinearity (0.8 to 0.9; Mason and Perreault 1991). Effect sizes remained small, and standard errors only showed minimal change from the main models, where a common consequence of multicollinearity is increased standard errors (Thompson et al. 2017).

## Discussion

In a large, longitudinal sample spanning ages 9 to 15, the present study characterized interindividual variability in trajectories of adolescent cortical development and examined how neighborhood characteristics relate to this variability. Individual differences in cortical thickness and surface area were associated with childhood neighborhood characteristics: those exposed to neighborhoods with higher levels of disadvantage had lower cortical thickness and surface area, and more rapid declines across adolescence. Greater opportunities in the neighborhood were associated with higher cortical thickness and surface area at baseline and a slower rate of change in these metrics over time. This suggests that early exposure to neighborhood disadvantage is associated with a faster pace of adolescent cortical development, while exposure to high-resource neighborhoods may foster a slower pace of maturation. Findings remained significant after controlling for family income-to-needs ratio and sex. The effect sizes for these associations were small overall, however. Our work highlights that in this US-based sample, adolescent cortical maturation is heterogeneous between individuals, and neighborhood contextual factors are related to this heterogeneity. This suggests that neighborhood environments may play a role in shaping cortical architecture across adolescence.

Our findings align with extensive research showing that neighborhood environments shape developmental outcomes (Leventhal and Brooks-Gunn 2000; Vanaken and Danckaerts 2018; Leventhal and Dupéré 2019; Abouzeid and Johnson 2024; Cai et al. 2024; Christensen et al. 2024; Ferrara et al. 2024; Morrel et al. 2025; Shoari et al. 2025; Thomas et al. 2025; Zhou et al. 2025). We found that greater neighborhood disadvantage was linked to reduced cortical thickness and surface area, consistent with cross-sectional studies (Parker et al. 2017; Hackman et al. 2021; Tomasi and Volkow 2021; Miller et al. 2022; Rakesh et al. 2022; Nolte et al. 2024). Our results extend cross-sectional work by showing accelerated declines in these measures across adolescence among youth exposed to higher disadvantage in childhood. These findings are in line with recent work in the ABCD study, indicating steeper reductions in cortical volume between ages 9 and 13 among those from disadvantaged neighborhoods (Thomas et al. 2025). However, other longitudinal work reports the opposite pattern, linking neighborhood deprivation to less cortical thickening across adolescence (Whittle et al. 2017) and a slower brain-predicted age trajectory (Rakesh et al. 2021a). These discrepancies may reflect differences in developmental timing, as early-life stress may influence the pace of cortical maturation differently across phases of adolescence (Vannucci et al. 2023). More long-term studies are needed to clarify how neighborhood contexts shape cortical developmental trajectories across the entire adolescent period.

Our finding that childhood neighborhood disadvantage was associated with accelerated cortical development aligns with evolutionary frameworks such as the stress acceleration hypothesis (Callaghan and Tottenham 2016), which postulates that stressful early-life environments can accelerate neurodevelopment (Tooley et al. 2021). It is possible that growing up in a disadvantaged neighborhood increases exposure to stressful stimuli, including community violence (Leventhal and Brooks-Gunn 2000; Evans and Kim 2010) or environmental pollutants (Trentacosta et al. 2016), accelerating structural brain development. However, it should be noted that our findings are inconsistent with other longitudinal work (Hanson et al. 2013; Hair et al. 2015; Whittle et al. 2017; McDermott et al. 2019; Barch et al. 2020, 2022; Rakesh et al. 2021a; Kalantar-Hormozi et al. 2023) and a recent review that suggests that socioeconomic disadvantage may be associated with a “slower” pace of cortical development (Rakesh et al. 2023a). Several of these studies examined subcortical and cortical volume, which may be contributing to the observed differences. Additionally, stressor characteristics, including dimensions of threat, deprivation, and predictability, may influence the pace of development differently (Colich et al. 2020; Roubinov et al. 2021; Ellis et al. 2022). It is also possible that supportive social environments can buffer the effects of stressful experiences or enable recalibration, if positive social environments are introduced after the stressful event (Laube and Fuhrmann 2020; Gee 2021; Rakesh et al. 2021b). Neighborhoods are complex ecological systems, comprising diverse stressors and supports, and it is thus likely that their effects on the pace of cortical development might differ depending on context-specific factors. Prospective longitudinal work is needed to explore how contextual factors interact and shape the relationship between neighborhood disadvantage and the pace of structural brain maturation over time.

We observed a relationship between increased opportunities in the neighborhood and higher cortical thickness and surface area, consistent with prior research linking positive environmental factors such as access to good quality schools, greenspace, and lower levels of air pollution with favorable neurodevelopmental outcomes (Herting et al. 2024; Li et al. 2025; Zhou et al. 2025). We additionally found that greater neighborhood opportunities were associated with attenuated reductions in cortical thickness and surface area, indicating slower development in these metrics across adolescence. Drawing on similar evolutionary principles to the stress acceleration hypothesis, it has been postulated that exposure to enriching environments may decelerate cortical maturation processes, potentially prolonging neuroplasticity (e.g., through increased synaptogenesis), supporting the development of more efficient adult cortical networks (Tooley et al. 2021). However, whether such developmental strategies are always beneficial is likely also context-dependent (Frankenhuis and Del Giudice 2012; Ellis et al. 2017). Thus, while we here demonstrate that high-resource neighborhoods are associated with a slower pace of adolescent cortical development, future research will be needed to explore the potential costs and benefits of these developmental patterns across different environmental contexts. Additionally, it is worth noting that when controlling for intracranial volume, associations between neighborhood disadvantage and educational opportunity and the pace of cortical thickness development were no longer statistically significant, suggesting that these specific relationships may be driven by differences in overall brain size rather than reflecting processes specific to cortical thickness development. Future research is needed to disentangle global versus region-specific associations between neighborhood characteristics and brain structure.

Our findings indicate that, when considered together, only neighborhood opportunities were uniquely associated with the slope of cortical thickness development, whereas both disadvantage and opportunity metrics were related to unique variance in baseline levels of cortical structure. This indicates that environmental enrichment may be more strongly associated with dynamic cortical thickness development across adolescence, while both disadvantage and opportunities are important for baseline structural differences. Given that prior literature has predominantly focused on the relationship between neighborhood disadvantage and neurodevelopmental outcomes, our findings emphasize the importance of using strength-based approaches, including opportunity, to characterize neighborhood contexts, beyond the narrow scope of disadvantage (Hyde et al. 2022; Choudhury et al. 2023; Polemiti et al. 2024; Rakesh et al. 2024; Lewis et al. 2025). Such studies can highlight how positive neighborhood characteristics may support neurodevelopment, offering an alternative to deficit-based approaches that focus solely on the negative impacts of adverse environments (DeJoseph et al. 2024; Zhou et al. 2025). Furthermore, given heterogeneities in neighborhood environments, including the potential for co-occurring positive (e.g., access to greenspace) and negative (e.g., lower educational opportunities) characteristics, it is likely that these factors interact with each other to influence individual variations in neurodevelopmental outcomes (Lewis et al. 2025). Future studies are needed to investigate how different neighborhood dimensions interact to shape cortical maturation across development.

We found individual differences in developmental trajectories of cortical thickness and surface area, in support of a growing body of evidence that structural brain development is heterogeneous between individuals (Becht and Mills 2020; Mills et al. 2021; Fuhrmann et al. 2022a; Bottenhorn et al. 2023, 2024; Carrick et al. 2025). Notably, while we observed individual variations in the intercept and slope of cortical thickness and surface area development, we did not identify distinct “subgroups” of cortical development, contrary to our hypotheses. Prior research has proposed several ways in which individual differences in structural brain development can manifest, including variations in intercepts and slopes, the age at which developmental milestones are reached, as well as the shape of trajectories (Olson et al. 2025). Here, although we see individual variation in the intercept and slope of development, the shapes of trajectories in the identified subgroups were parallel. It is possible that individual differences in cortical maturation are distributed continuously and do not manifest as discrete subgroups of development with divergent trajectory shapes. As developmental samples with repeated timepoints emerge, future research can employ sophisticated statistical approaches (e.g., nonlinear mixed-effects modeling; Fuhrmann et al. 2022a; Carrick et al. 2025) to characterize the nature of individual differences in cortical maturation across adolescence.

This study is strengthened by its preregistered design and use of a large longitudinal sample, enabling us to investigate the association between multifaceted neighborhood environments and individual differences in cortical development over time. At the same time, our findings should be considered in light of several sample-related limitations. First, given that neighborhood characteristics are dependent upon country-specific factors, the following findings may be limited in generalizability beyond the US context. Second, prior research indicates that attrition in the ABCD study is not independent of ethnicity, nor neighborhood characteristics (Rakesh et al. 2025). This may limit the generalizability of findings. However, it is worth noting that, while participants with two or more timepoints tended to be from less disadvantaged neighborhoods, neighborhood opportunity metrics differed only marginally between those with one timepoint and those with two or more (Supplementary Table 1). Third, at the time of analysis, ABCD data release 5.1 only had partial data available from the third imaging timepoint, thus limiting the sample size of participants with three available scans. Future analyses using subsequent releases of ABCD data may reveal different patterns. It is further worth noting that the effect sizes observed in the present study were small in magnitude. In large-scale studies such as ABCD, power is high, and therefore, small associations can reach statistical significance, limiting the interpretability of *P*-values alone (Dick et al. 2025). However, evidence from large brain-wide association studies, including ABCD, indicates that reproducible associations typically have small effects (Marek et al. 2022), and researchers have proposed that small effects can be meaningful when applied to the population level (Funder and Ozer 2019). Given the limited longitudinal work that has examined cortical development in relation to neighborhood characteristics, future work will be needed to benchmark the observed effect sizes and clarify the extent to which they are meaningful.

Beyond data-related limitations, there are several other limitations to consider. While we demonstrate the importance of objective measures of neighborhood disadvantage and opportunity for structural brain development, research suggests that subjective perceptions of neighborhood features, for example, perceived neighborhood safety, might also influence neurodevelopmental outcomes. Future studies would benefit from incorporating both subjective and objective indicators of neighborhood contexts in their studies. Furthermore, while we here link neighborhood contextual factors to interindividual variation in structural brain development, future research will need to investigate whether and how these associations are relevant for later-life outcomes, such as cognitive functioning and mental health. Neighborhood characteristics have been linked to a range of emotional and behavioral difficulties, as well as functioning across multiple cognitive domains (Taylor et al. 2020; Li et al. 2023; Rakesh et al. 2025; Shoari et al. 2025). Detailed investigations examining the mediating influence of brain structure in the relationship between neighborhoods and cognitive and mental health outcomes are warranted. Moreover, while we discuss evolutionary theories such as the stress acceleration hypothesis as a possible explanation for the relationship between neighborhood context and variations in structural brain development, we do not examine whether neighborhoods with higher disadvantage and lower opportunity are linked to heightened stress exposure. Future research should explicitly explore the mediating effect of stress in this relationship. Finally, while the present study provides insight into the relationships between neighborhood characteristics and global metrics of cortical development, it is limited by its lack of investigation into the specific cortical regions that may be implicated in this association. Future research will benefit from hypothesis-driven, region-specific approaches, potentially identifying specific cortical areas that are sensitive to neighborhood contexts. This can more precisely elucidate neural mechanisms linking neighborhood environmental factors to cognitive and mental health outcomes.

## Conclusion

Overall, our work elucidates interindividual variability in trajectories of cortical maturation and provides unique insight into the role of neighborhoods in shaping this variability. We here find evidence that neighborhoods characterized by greater disadvantage are associated with lower levels and faster rates of decline in cortical thickness and surface area across adolescence. Those exposed to high-opportunity neighborhoods had greater cortical thickness and surface area and a slower pace of change. Future longitudinal work is needed to examine how multifaceted neighborhood contexts, including both positive and negative features, interact to influence interindividual variability in cortical development, and how these relationships relate to later-life outcomes, including mental health and cognitive functioning.

## Supplementary Material

Carrick_et_al_Supplementary_bhag034

## Data Availability

This study harnessed data from the ABCD study (https://abcdstudy.org/), hosted on the National Institute of Mental Health Data Archive (https://nda.nih.gov/).
