# Supplementary material for: Individual differences in adolescent cortical development are associated with neighborhood characteristics: Longitudinal findings from the ABCD study
Source: Cereb Cortex. 2026 Apr 7;36(4):bhag034. doi: 10.1093/cercor/bhag034 (PMC13064848; doi:10.1093/cercor/bhag034)
Supplement: Carrick_et_al_Supplementary_bhag034 [file carrick_et_al_supplementary_bhag034.docx]

**Supplementary Materials:**

Individual differences in adolescent cortical development are associated with neighbourhood characteristics: Longitudinal findings from the ABCD study

**Authors:** Chloe Carrick*^a^, Divyangana Rakesh^b^, Lea Michel^c^, Kathryn Bates^a^, Delia Fuhrmann^a^

**Affiliations:** ^a^Department of Psychology, Institute of Psychiatry, Psychology, and Neuroscience, King’s College London, London, United Kingdom; ^b^Neuroimaging Department, Institute of Psychiatry, Psychology, and Neuroscience, King’s College London, London, United Kingdom; ^c^Cognitive Neuroscience Department, Radboud University Medical Center, Nijmegen, The Netherlands

***Corresponding author:**

Chloe Carrick

Department of Psychology

Kings College London

Great Maze Pond, London, SE1 9RT

[chloe.carrick@kcl.ac.uk](mailto:chloe.carrick@kcl.ac.uk)

**ORCID ID:** 0009-0002-6219-4341

**Supplementary Table 1A**

| **Demographic Characteristic** | **T1 (*N* = 11,270)** | **T2 (*N* = 7,896)** | **T3 (*N* = 3,000)** |
| --- | --- | --- | --- |
| **Sex** | ***N* (%)** | ***N* (%)** | ***N* (%)** |
| Male | 5841 (51.83%) | 4192 (53.01%) | 1571 (52.37%) |
| Female | 5426 (48.15%) | 3638 (46.07%) | 1395 (46.50%) |
| Intersex-Male | <10 | <10 | <10 |
| **Ethnicity** | ***N (%)*** | ***N (%)*** | ***N (%)*** |
| White | 5900 (53.35%) | 4190 (53.06%) | 1626 (54.20%) |
| Hispanic | 2301 (20.42%) | 1446 (18.31%) | 567 (18.90%) |
| Black | 1656 (14.69%) | 1004 (12.72%) | 290 (9.67%) |
| Asian | 237 (2.10%) | 141 (1.78%) | 62 (2.06%) |
| Other | 1174 (10.42%) | 782 (9.90%) | 298 (9.93%) |
| **Neighbourhood characteristics** | **M (SD) [range]** | **M (SD) [range]** | **M (SD) [range]** |
| Neighbourhood disadvantage | -0.01(0.84)[-1.49 – 3.92] | -0.03(0.82)[-1.42-3.90] | -0.08(0.76)[-1.36-3.90] |
| Educational opportunity | 60.41(29.69)[1-100] | 60.49(29.50)[1,100] | 61.45(29.11)[1-100] |
| Health/environmental opportunity | 58.73(30.12)[1-100] | 58.99(29.83)[1-100] | 59.03(28.38)[1-100] |
| **Income-to-needs ratio** | 3.72(2.46)[ 0.03 - 12.32] | 3.72( 2.40)[ 0.05- 12.32] | 3.8( 2.42)[0.06-12.32] |

Participant demographic information at each timepoint.

*Note.* M = mean. SD = standard deviation. T1, T2, T3 = Timepoints 1,2, and 3.

**Supplementary Table 1B.**

| **Demographic Characteristic** | **1 timepoint (*N* = 3558)** | | $\boldsymbol{\geq}$**2 timepoints (*N* = 8081)** |
| --- | --- | --- | --- |
| **Sex** | | ***N* (%)** | ***N* (%)** |
| Male | | 1744 (49.01%) | 4273(52.88%) |
| Female | | 1767(49.66%) | 3780(46.78%) |
| Intersex-Male | | < 10 | <10 |
| **Ethnicity** | | ***N (%)*** | ***N (%)*** |
| White | | 1460 (41.1%) | 4440 (55.8%) |
| Hispanic | | 791 (22.3%) | 1510 (19.0%) |
| Black | | 618 (17.4%) | 1038 (13.0%) |
| Asian | | 84 (2.36%) | 153 (1.92%) |
| Other | | 355 (9.98%) | 819 (10.3%) |
| **Neighbourhood characteristics** | | **M (SD) [range]** | **M (SD) [range]** |
| Neighbourhood disadvantage | | 0.09(0.9)[-1.49 - 3.92] | -0.04(0.81)[-1.42 – 3.90] |
| Educational opportunity | | 59.37(30.32)[1-100] | 60.8(29.40)[1-100] |
| Health/environmental opportunity | | 57.55(31.14)[1-100] | 59.2(29.70)[1-100] |
| **Income-to-needs ratio** | | 3.63( 2.58)[ 0.03- 12.32] | 3.76( 2.41)[0.05- 12.30] |

Participant demographic information for those with differing numbers of timepoints.

*Note.* M = mean. SD = standard deviation. 1 timepoint = participants with one neuroimaging timepoint only. $\boldsymbol{\geq}$2 timepoints = participants with 2 or more timepoints across the study period

**Supplementary Table 2**

Number and percentage of participants missing per variable

| **Variable** | ***N* (%)** |
| --- | --- |
| Sex | 72 (0.62) |
| Ethnicity | 371 (3.19) |
| Neighbourhood Disadvantage | 992 (8.52) |
| Educational Opportunity | 1407 (12.09) |
| Health/Environmental Opportunity | 1407 (12.09) |
| Income-to-needs ratio | 1491 (12.81) |
|  |  |
| CT/SA: |  |
| Timepoint 1 | 369(3.17) |
| Timepoint 2 | 3743 (32.16) |
| Timepoint 3 | 8639 (74.22) |
| *Note.* CT = cortical thickness. SA = cortical surface area. | |

**Supplementary Table 3.**

Area Deprivation Index Items included in Neighbourhood Disadvantage measure.

| Percentage of population aged >/= 25 with at least a high school diploma |
| --- |
| Median family income |
| Income disparity |
| Percentage of home-owners |
| Percentage of labour force aged > 16 and unemployed |
| Percentage of families below poverty level |
| Percentage of families below 138% of poverty threshold |
| Percentage of single parent housing |
| Percentage of homes without a motor vehicle |

*Note*. These nine items assess poverty level, income, employment, education, the percentage of single-parent households, homeowners, and homes without a motor vehicle in the participant’s neighbourhood. Thus, these items reflect disadvantage constructs that are not dependent on location-based differences in cost-of-living (e.g., mortgage rates) or outdated disadvantage indicators (e.g., percentage of homes without a telephone; Taylor et al., 2020).

**Supplementary Table 4.**

Percentage of participants in each subgroup, and BIC values, for growth mixture models and latent class growth analyses, fit to cortical thickness and surface area development.

| **Growth mixture models** | | | | | | |
| --- | --- | --- | --- | --- | --- | --- |
| **CT** | **Model** | **% S1** | **% S2** | **% S3** | **% S4** | **BIC** |
|  | 1 subgroup | 100 |  |  |  | 38853.68 |
|  | 2 subgroups | 1.45 | 98.55 |  |  | 38387.51 |
|  | 3 subgroups | 0.88 | 77.02 | 22.10 |  | 38685.62 |
|  | 4 subgroups | 1.65 | 0.77 | 97.43 | 0.15 | 38076.58 |
| **SA** | **Model** | **% S1** | **% S2** | **% S3** | **% S4** | **BIC** |
|  | 1 subgroup | 100 |  |  |  | 50822.85 |
|  | 2 subgroups | 1.22 | 98.78 |  |  | 50214.26 |
|  | 3 subgroups | 1.20 | 15.95 | 82.85 |  | 50178.67 |
|  | 4 subgroups | 2.58 | 48.16 | 28.60 | 20.66 | 50220.50 |
| **Latent class growth analyses** | | | | | | |
| **CT** | **Model** | **% S1** | **% S2** | **% S3** | **% S4** | **BIC** |
|  | 1 subgroup | 100 |  |  |  | 52465.88 |
|  | 2 subgroups | 50.61 | 49.39 |  |  | 46533.56 |
|  | 3 subgroups | 20.84 | 22.61 | 56.55 |  | 43195.61 |
|  | 4 subgroups | 9.07 | 40.42 | 39.20 | 11.30 | 41214.81 |
| **SA** | **Model** | **% S1** | **% S2** | **% S3** | **% S4** | **BIC** |
|  | 1 subgroup | 100 |  |  |  | 84393.68 |
|  | 2 subgroups | 56.01 | 43.99 |  |  | 76492.75 |
|  | 3 subgroups | 49.60 | 18.58 | 31.83 |  | 71223.82 |
|  | 4 subgroups | 17.23 | 38.51 | 33.51 | 10.76 | 67296.82 |

*Note*. % S1-S4 = percentage of participants in subgroups 1-4. At least one subgroup had < 5% for each GMM fit to cortical thickness and surface area development. CT = cortical thickness. SA = cortical surface area. BIC = Bayesian Information Criterion

**Supplementary Table 5.**

Adjusted analyses: Latent growth models including sex, scanner type, and income-to-needs ratio as covariates in regression paths.

| **Neighbourhood disadvantage** | | | | | | |  |
| --- | --- | --- | --- | --- | --- | --- | --- |
| **CT** | **Regression path** | **Variable** | **Estimate** | **SE** | **z-value** | ***p*-value** | **Std. Est** |
|  | Slope~ ND + Sex + Scanner + INR | ND | -0.03 | 0.01 | -2.19 | 0.03 | -0.10 |
|  | Intercept ~ ND + Sex + Scanner + INR | ND | -0.09 | 0.01 | -8.85 | <.001 | -0.11 |
| **SA** | **Regression path** | **Variable** | **Estimate** | **SE** | **z-value** | ***p*-value** | **Std. Est** |
|  | Slope~ ND + Sex + Scanner + INR | ND | -0.002 | 0.001 | -2.79 | 0.01 | -0.10 |
|  | Intercept ~ ND + Sex + Scanner + INR | ND | -0.02 | 0.001 | -13.30 | <.001 | -0.13 |
| **Educational opportunity** | | | | | | |  |
| **CT** | **Regression path** | **Variable** | **Estimate** | **SE** | **z-value** | ***p*-value** | **Std. Est** |
|  | Slope~ EO + Sex + Scanner + INR | EO | 0.001 | 0.0002 | 2.81 | 0.01 | 0.11 |
|  | Intercept ~ EO + Sex + Scanner + INR | EO | 0.002 | 0.0003 | 7.39 | <.001 | 0.09 |
| **SA** | **Regression path** | **Variable** | **Estimate** | **SE** | **z-value** | ***p*-value** | **Std. Est** |
|  | Slope~ EO + Sex + Scanner + INR | EO | 0.00005 | 0.00002 | 2.77 | 0.01 | 0.09 |
|  | Intercept ~ EO + Sex + Scanner + INR | EO | 0.0004 | 0.00004 | 10.99 | <.001 | 0.11 |
| **Health /Environmental opportunity** | | | | | | |  |
| **CT** | **Regression path** | **Variable** | **Estimate** | **SE** | **z-value** | ***p*-value** | **Std. Est** |
|  | Slope~ HO + Sex + Scanner +INR | HO | 0.001 | 0.0003 | 3.71 | <.001 | 0.15 |
|  | Intercept ~ HO + Sex + Scanner +INR | HO | 0.002 | 0.0003 | 8.10 | <.001 | 0.09 |
| **SA** | **Regression path** | **Variable** | **Estimate** | **SE** | **z-value** | ***p*-value** | **Std. Est** |
|  | Slope~ HO + Sex + Scanner + INR | HO | 0.00004 | 0.00002 | 2.75 | 0.01 | 0.08 |
|  | Intercept ~ HO + Sex + Scanner + INR | HO | 0.0004 | 0.00004 | 10.67 | <.001 | 0.10 |
| *Note*. Latent growth model regression paths examining the association between neighbourhood characteristics and cortical thickness and surface area development, with sex, scanner type, and income-to-needs ratio included as covariates. CT = cortical thickness. SA = cortical surface area. ND = neighbourhood disadvantage. EO = neighbourhood educational opportunity. HO = neighbourhood health and environmental opportunity. INR = income-to-needs ratio. SE = standard error. Std. Est = Standardised Estimate. | | | | | | | |

| **Supplementary Table 6.**  Latent growth models adjusted for the influence of intracranial volume | | | | | | | | |
| --- | --- | --- | --- | --- | --- | --- | --- | --- |
| **Neighbourhood disadvantage** | | | | | | | | |
| **CT** | **Regression path** | **Estimate** | **SE** | **z-value** | ***p*-value** | **Std. Est** | |  |
|  | Slope~ ND | -0.02 | 0.01 | -1.84 | 0.07 | -0.07 | |  |
|  | Intercept ~ ND | -0.11 | 0.01 | -12.38 | <.001 | -0.13 | |  |
| **SA** | **Regression path** | **Estimate** | **SE** | **z-value** | ***p*-value** | **Std. Est** | |  |
|  | Slope~ ND | -0.002 | 0.001 | -3.14 | 0.002 | -0.08 | |  |
|  | Intercept ~ ND | -0.02 | 0.001 | -20.36 | <.001 | -0.20 | |  |
| **Educational opportunity** | | | | | | | | |
| **CT** | **Regression path** | **Estimate** | **SE** | **z-value** | ***p*-value** | **Std. Est** | |  |
|  | Slope~ EO | 0.001 | 0.0003 | 1.94 | 0.05 | 0.07 | |  |
|  | Intercept ~ EO | 0.003 | 0.0003 | 11.16 | <.001 | 0.12 | |  |
| **SA** | **Regression path** | **Estimate** | **SE** | **z-value** | ***p*-value** | **Std. Est** | |  |
|  | Slope~ EO | 0.00006 | 0.00002 | 3.33 | 0.001 | 0.08 | |  |
|  | Intercept ~ EO | 0.001 | 0.00003 | 15.94 | <.001 | 0.17 | |  |
| **Health /Environmental opportunity** | | | | | | | | |
| **CT** | **Regression path** | **Estimate** | **SE** | **z-value** | ***p*-value** | **Std. Est** | |  |
|  | Slope~ HO | 0.001 | 0.0003 | 2.99 | 0.003 | 0.11 | |  |
|  | Intercept ~ HO | 0.003 | 0.0003 | 11.63 | <.001 | 0.13 | |  |
| **SA** | **Regression path** | **Estimate** | **SE** | **z-value** | ***p*-value** | **Std. Est** | |  |
|  | Slope~ HO | 0.0001 | 0.00002 | 4.00 | <.001 | 0.10 | |  |
|  | Intercept ~ HO | 0.001 | 0.0003 | 16.02 | <.001 | 0.17 | |  |
| *Note*. Latent growth model regression paths examining the association between neighbourhood characteristics and cortical thickness and surface area development, adjusting for the influence of intracranial volume. Highlighted in red are the two associations that no longer reached statistical significance. CT = cortical thickness. SA = cortical surface area. ND = neighbourhood disadvantage. EO = neighbourhood educational opportunity. HO = neighbourhood health and environmental opportunity. SE = standard error. Std. Est = Standardised Estimate. | | | | | | |  |  |

**Supplementary Table 7.**

Specificity analyses: Latent growth models including all neighbourhood characteristics in regression paths.

| **Specificity Analysis** | | | | | | |  |
| --- | --- | --- | --- | --- | --- | --- | --- |
| **CT** | **Regression path** | **Variable** | **Estimate** | **SE** | **z-value** | ***p*-value** | **Std. Est** |
|  | Slope ~ ND + EO + HO | ND | 0.004 | 0.02 | 0.27 | 0.79 | 0.02 |
|  |  | EO | 0.0003 | 0.0004 | 0.85 | 0.39 | 0.04 |
|  |  | HO | 0.001 | 0.0004 | 2.68 | 0.01 | 0.14 |
|  | Intercept ~ ND + EO + HO | ND | -0.06 | 0.02 | -4.31 | <.001 | -0.07 |
|  |  | EO | 0.001 | 0.0004 | 3.19 | 0.001 | 0.05 |
|  |  | HO | 0.001 | 0.0004 | 3.27 | 0.001 | 0.05 |
| **SA** | **Regression path** | **Variable** | **Estimate** | **SE** | **z-value** | ***p*-value** | **Std. Est** |
|  | Slope ~ ND + EO + HO | ND | 0.0001 | 0.001 | 0.18 | 0.86 | 0.01 |
|  |  | EO | 0.00004 | 0.00002 | 2.05 | 0.04 | 0.09 |
|  |  | HO | 0.00004 | 0.00002 | 1.97 | 0.05 | 0.09 |
|  | Intercept ~ ND + EO + HO | ND | -0.02 | 0.002 | -9.23 | <.001 | -0.15 |
|  |  | EO | 0.0002 | 0.0001 | 3.87 | < .001 | 0.06 |
|  |  | HO | 0.0001 | 0.0001 | 2.17 | 0.03 | 0.03 |
| **Adjusted Specificity Analysis** | | | | | | |  |
| **CT** | **Regression path** | **Variable** | **Estimate** | **SE** | **z-value** | ***p*-value** | **Std. Est** |
|  | Slope ~ ND + EO + HO + sex + scanner + INR | ND | 0.01 | 0.02 | 0.52 | 0.60 | 0.03 |
|  |  | EO | 0.0004 | 0.0004 | 1.08 | 0.28 | 0.05 |
|  |  | HO | 0.001 | 0.0004 | 2.58 | 0.01 | 0.13 |
|  | Intercept ~ ND + EO + HO + sex + scanner + INR | ND | -0.06 | 0.02 | -3.74 | <.001 | -0.07 |
|  |  | EO | 0.001 | 0.0004 | 1.66 | 0.10 | 0.03 |
|  |  | HO | 0.001 | 0.0004 | 2.27 | 0.02 | 0.07 |
| **SA** | **Regression** | **Variable** | **Estimate** | **SE** | **z-value** | ***p*-value** | **Std. Est** |
|  | Slope ~ ND + EO + HO + sex + scanner + INR | ND | -0.001 | 0.001 | -0.68 | 0.50 | -0.03 |
|  |  | EO | 0.00003 | 0.00002 | 1.50 | 0.13 | 0.06 |
|  |  | HO | 0.00002 | 0.00002 | 0.88 | 0.38 | 0.04 |
|  | Intercept ~ ND + EO + HO + sex + scanner + INR | ND | -0.01 | 0.002 | -6.27 | <.001 | -0.09 |
|  |  | EO | 0.0001 | 0.0001 | 3.28 | 0.001 | 0.04 |
|  |  | HO | 0.0001 | 0.0001 | 1.86 | 0.06 | 0.02 |

*Note*. Adjusted specificity analysis additionally controls for sex, scanner type, and income-to-needs ratio in models. CT = cortical thickness. SA = cortical surface area. ND = neighbourhood disadvantage, EO = educational opportunity, HO = health and environmental opportunity. INR = income-to-needs ratio. Std. Est = Standardised Estimate.


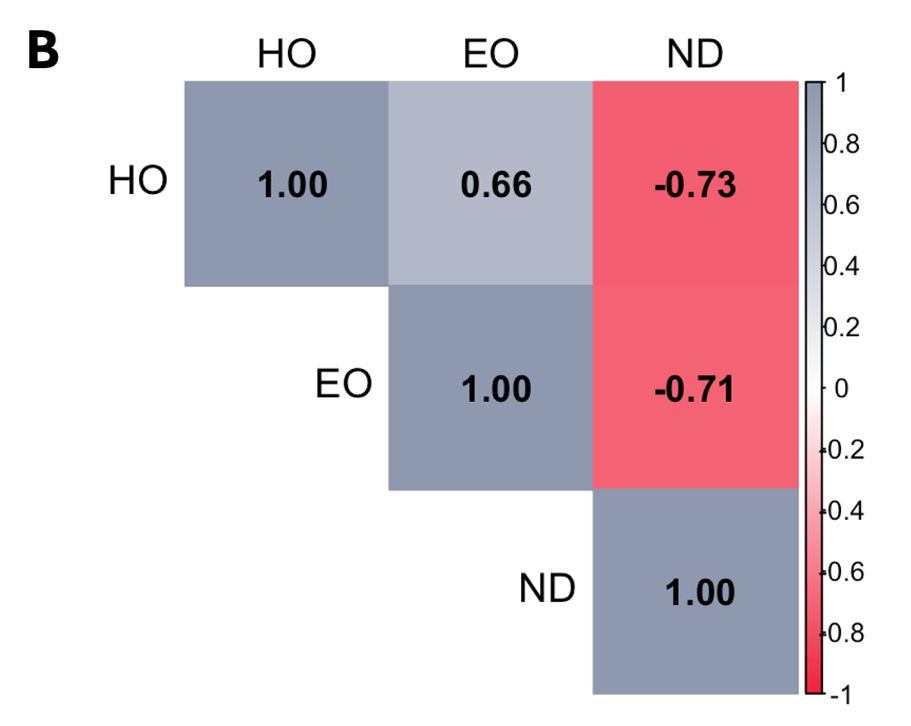

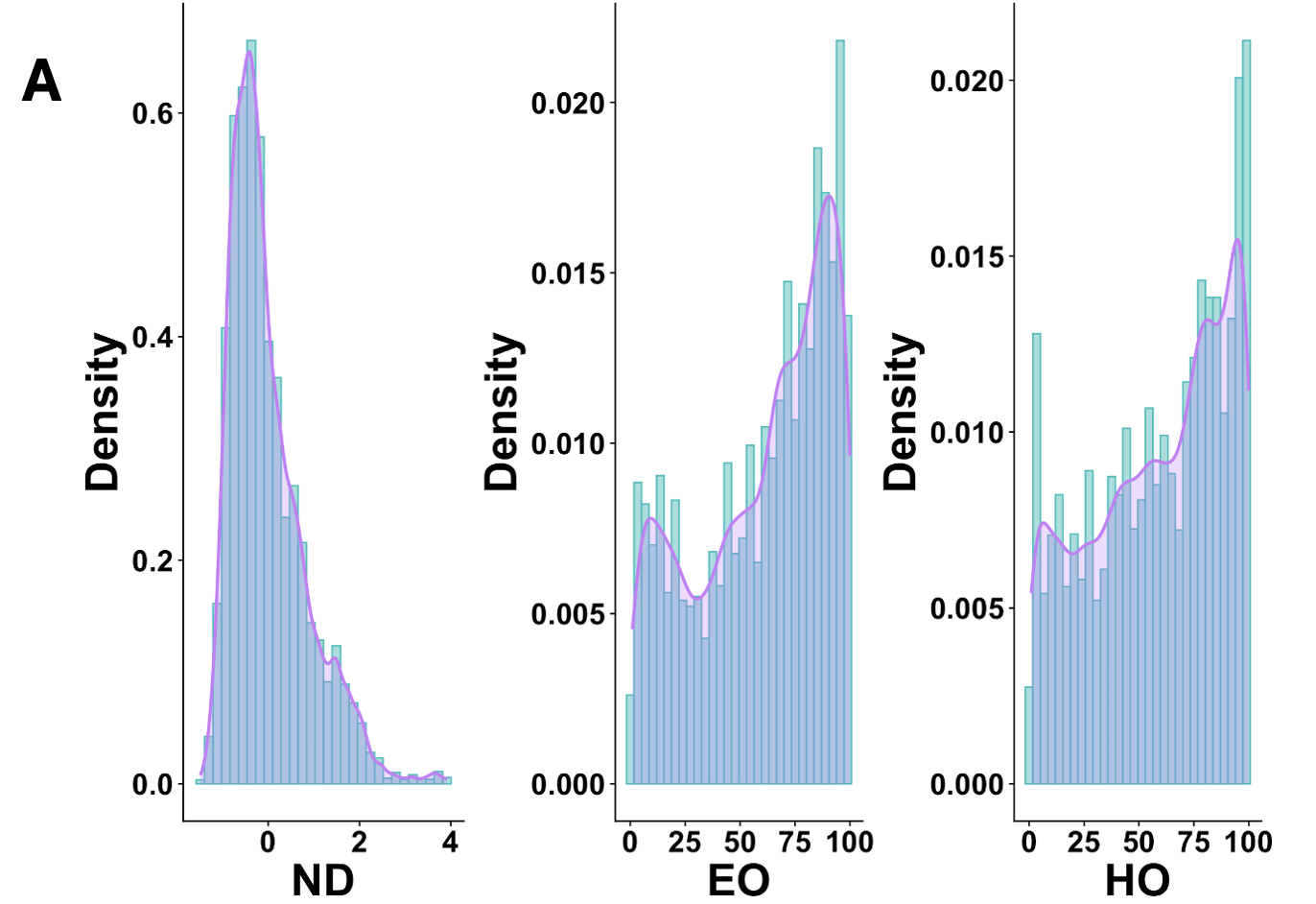


**Supplementary Figure 1.** A) Density plots of neighbourhood characteristics. B) Pearson’s bivariate correlations between neighbourhood characteristics. ND = neighbourhood disadvantage. EO = educational opportunity. HO = health/environmental opportunity.


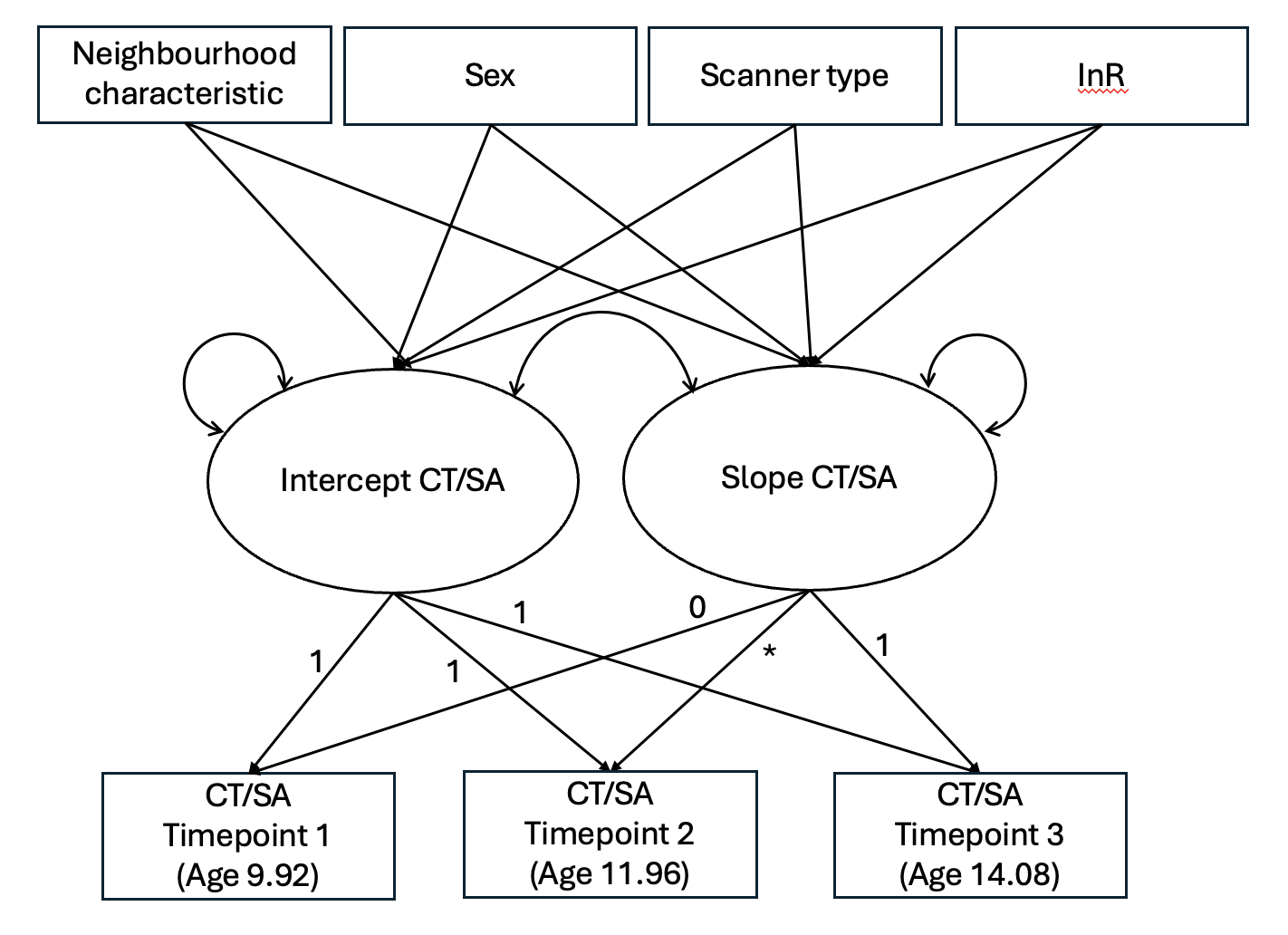


**Supplementary Figure 2.** Schematic path diagram depicting latent growth models with neighbourhood characteristics as predictors of the intercept and slope of cortical development, adjusted for the confounding effects of sex, scanner type and income-to-needs ratio (InR). Average age is presented at each timepoint. Double-headed arrows represent the slope and intercept variances and covariances. Numbers represent the factor loadings. * = factor loading was freely estimated. CT = cortical thickness. SA = cortical surface area. InR = Income-to-needs ratio.


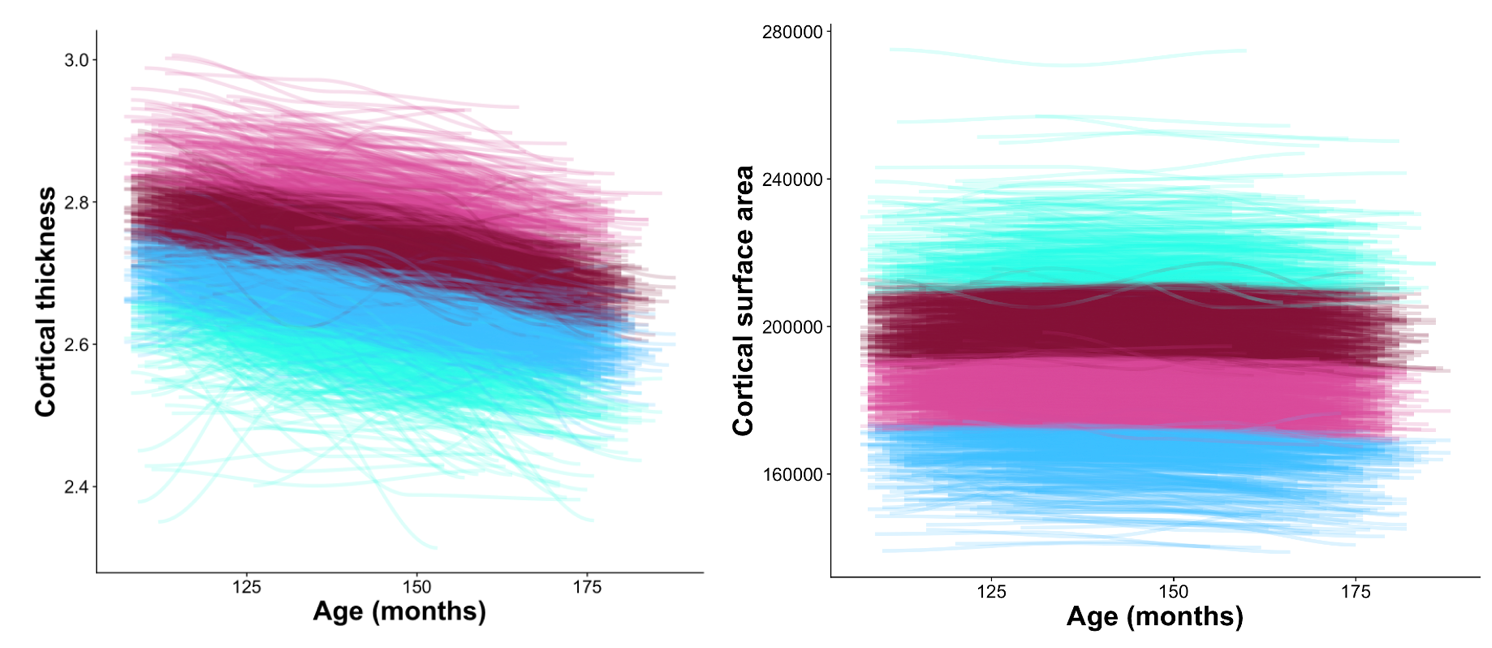


**Supplementary Figure 3.** Trajectories of subgroups cortical thickness and surface area development derived from latent class growth analyses. Spaghetti plots indicate individual participant trajectories, with different colours indicating identified subgroups of cortical development. Presented are the models with four subgroups, which had the lowest BIC values (see Supplementary Table 4).


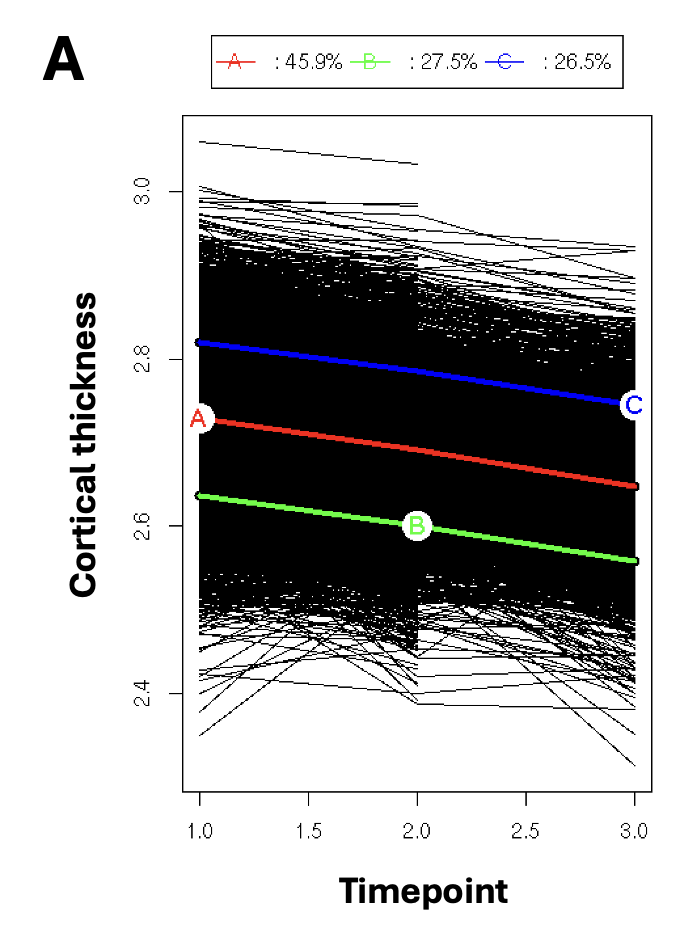

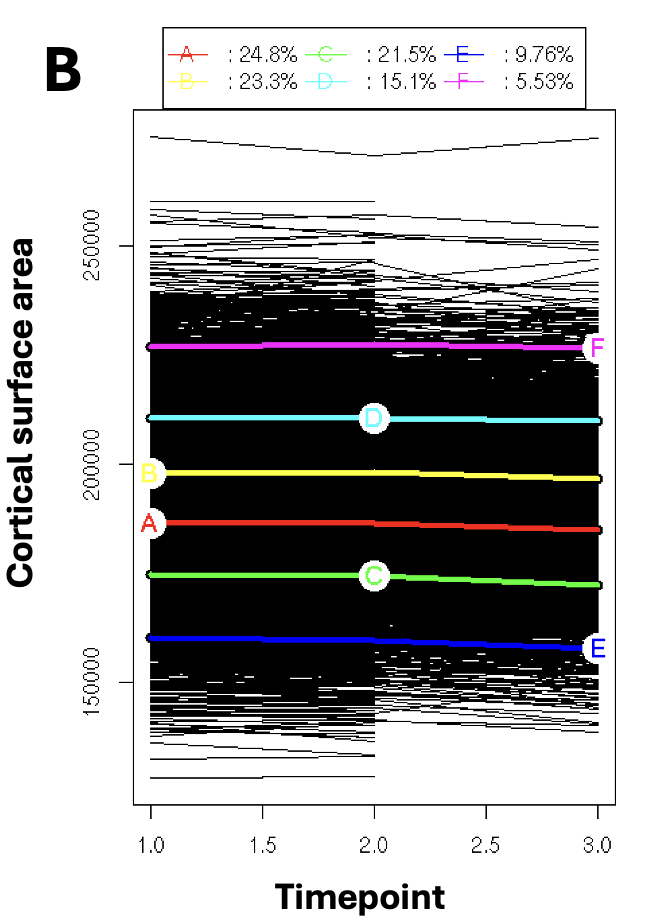


**Supplementary Figure 4**. Trajectories of subgroups of cortical thickness (A) and cortical surface area development (B) identified using k-means clustering. Mean trajectories in each subgroup (presented in coloured lines) are overlaid on spaghetti plots of individual participant trajectories (in black). Presented are the 3-cluster solution for cortical thickness, and the 6-cluster solution for surface area, which were the optimal fitting solutions according to the default kml quality criterion.
